# COVID-19 vaccines protect children of all ages

**DOI:** 10.1172/JCI164102

**Published:** 2022-09-01

**Authors:** Justin Z. Amarin, Haya Hayek, Natasha B. Halasa

**Affiliations:** Department of Pediatrics, Vanderbilt University Medical Center, Nashville, Tennessee, USA.

As of June 30, 2022, there have been 87,298,343 cases of coronavirus disease 2019 (COVID-19) reported in the United States, of which approximately 17.4% occurred in children 0–17 years old (1). So far in 2022, individuals 0–19 years old have accounted for 23.6% of cases, a considerable increase, from 14.4%, throughout 2020 (Figure 1). The increasing case burden in children corresponds with differential vaccine access and uptake compared with adults, of whom the vast majority received an mRNA COVID-19 vaccine (Pfizer-BioNTech or Moderna; ref. 1). The first Emergency Use Authorization (EUA) for Pfizer-BioNTech was issued on December 11, 2020, by the FDA, and it was the only available option for individuals 16 years and older (Figure 1 and ref. 2). It was not until May 10, 2021, almost 5 months later, that the FDA expanded the EUA for Pfizer-BioNTech to include adolescents 12–15 years old (Figure 1 and ref. 3). Some 6 months after that, on November 3, 2021, the US FDA amended the EUA to include children 5–11 years old (Figure 1 and refs. 1, 4). More than 1.5 years after the first EUA, on June 17, 2022, the Pfizer-BioNTech and Moderna COVID-19 vaccines were authorized for use in children 6 months and older (Figure 1 and ref. 5). Notably, Moderna had only been authorized for use in adults 18 years and older prior to the most recent EUA amendment (5).

Despite the CDC recommendation that everyone 6 months and older get vaccinated against COVID-19, vaccine uptake has been suboptimal in children compared with adults; 63.4% of children 5–11 years old were unvaccinated as of June 30, 2022, as opposed to 30.0% of adolescents 12–17 years old and 10.1% of adults at aged 18 years and older (1). According to a systematic review and meta-analysis of 44 studies that included 317,055 parents, only 60.1% intended to vaccinate their children against COVID-19 (6). Although many factors contribute to parental vaccine hesitancy, chief among them may be the perception that the risks of SARS-CoV-2 exposure in children are trivial and do not warrant prevention — a perception that is not substantiated. Other factors contributing to vaccine hesitancy include the risk of adverse events, such as myocarditis and pericarditis, and the perception that there is limited evidence from COVID-19 vaccine trials in children. Here we provide an overview of current evidence on the risks of SARS-CoV-2 infection balanced against the risks and benefits of COVID-19 vaccines in children.

## Risks of SARS-CoV-2 infection

Though generally more susceptible to respiratory viruses, children are less susceptible to SARS-CoV-2, which causes COVID-19. Early in the pandemic, many studies showed that SARS-CoV-2 affected children less frequently and severely than it did adults; roughly one in five children (a conservative estimate) with evidence of SARS-CoV-2 infection is asymptomatic (7). However, SARS-CoV-2 can cause severe illness in children — 1,604 COVID-19–related deaths have occurred in children 0–17 years old as of June 30, 2022 (1). Some children with acute COVID-19, especially those with underlying medical conditions, become critically ill and require intensive care, including mechanical ventilation, vasopressor support, and extracorporeal membrane oxygenation (8). In addition, some experience other complications, including multisystem inflammatory disorder in children (MIS-C) and long COVID-19 (9).

## MIS-C following SARS-CoV-2 infection

MIS-C is a rare, potentially life-threatening hyperinflammatory condition in individuals under 21 years old that typically develops within weeks of SARS-CoV-2 infection. As of June 30, 2022, the number of MIS-C cases in the United States stands at 8,639, with 70 deaths reported (1). Left-ventricular dysfunction and shock are typical of MIS-C, and potential long-term sequelae, including coronary artery aneurysms, carry high risk of morbidity (9). Although therapies are available for MIS-C, the condition is vaccine preventable (9–11). A two-dose series of Pfizer-BioNTech was 91% (95% CI, 78%–97%) effective against MIS-C in a study of US adolescents and 94% (95% CI, 55%–99%) effective in a study of children and adolescents in Denmark (10, 11). In the former study, none of the fully vaccinated adolescents with MIS-C required respiratory or cardiovascular support, as opposed to 39% of their unvaccinated counterparts (11). Therefore, COVID-19 vaccines effectively protect children from MIS-C and lessen illness severity in cases of breakthrough infection.

## MIS-C following COVID-19 vaccination

Some authors have reported cases of an exceedingly rare, MIS-C–like hyperinflammatory condition following COVID-19 vaccination in children with no serologic evidence of antecedent SARS-CoV-2 infection (12, 13). According to a multicenter study in the United States, the rate of MIS-C in individuals 12–20 years old with a history of vaccination and no serologic evidence of SARS-CoV-2 infection was 0.3 per 1,000,000 vaccinees (12). In comparison, the overall US rate of MIS-C following SARS-CoV-2 infection was 224 per 1,000,000 infections in children 11–15 years old and 164 per 1,000,000 infections in individuals 16–20 years old (12). According to another population-based study in France, the rate of MIS-C in children 12–17 years old with a history of vaccination and no serologic evidence of SARS-CoV-2 infection was 1.0 per 1,000,000 doses. In the same study, the authors estimated the rate of MIS-C following SARS-CoV-2 infection to be 113 per 1,000,000 infected children 12–17 years old (13). Notwithstanding the lack of causal evidence supporting a link between vaccination and MIS-C, the rate of MIS-C following SARS-CoV-2 infection is up to several-hundred-fold higher than the rate of MIS-C following vaccination. Therefore, the benefit of COVID-19 vaccines in preventing infection-induced MIS-C far outweighs the risk of vaccine-induced MIS-C.

## Other risks of COVID-19 vaccination

In addition to the predictable and common side effects of COVID-19 vaccines — such as injection site reactions, fever, and fatigue — other well-cited risks associated with COVID-19 vaccines are myocarditis and pericarditis. The risks appear to be highest in adolescent and young adult male individuals following vaccination with the Pfizer-BioNTech and Moderna vaccines, respectively (5). According to the aforementioned population-based study in France, the combined rate of myocarditis and pericarditis was 12.6 per 1,000,000 doses in children 12–17 years old (13). Therefore, these conditions are exceedingly rare compared with the risk of MIS-C following SARS-CoV-2 infection — which is itself a rare complication — in the same population. In addition, the conditions resolve rapidly following conservative management, and long-term sequelae have not been reported (5, 13).

## SARS-CoV-2 variants of concern

SARS-CoV-2 variants of concern have continually occurred and superseded each other since the beginning of the pandemic. As of June 30, 2022, Omicron is the predominant variant worldwide, with the BA.5, BA.2.12.1, BA.4, and BA.2 lineages accounting for all cases in the United States (1). The transition from one variant of concern to the next may alter the balance between the benefits and risks of the COVID-19 vaccines in children. For example, the Pfizer-BioNTech vaccine was highly effective against the Delta variant but only modestly effective against the Omicron variant in children 5–17 years old. However, vaccination prevented critical illness (i.e., requiring life support or culminating in death) caused by either variant and is therefore indicated (14, 15). In addition, the Pfizer-BioNTech vaccine was highly effective against MIS-C when Delta was predominant (10, 11). However, MIS-C has been occurring less frequently during the Omicron wave. For the first time since the CDC began tracking case reports of MIS-C, the 7-day moving average of new cases dropped to 0 in June 2022 (1). A population-based study in Denmark showed that the risk of MIS-C following SARS-CoV-2 infection was substantially lower during the Omicron than the Delta wave. Nevertheless, the authors also showed that the risk of MIS-C following SARS-CoV-2 infection during the Omicron wave was lower in vaccinated children and adolescents (16). All in all, COVID-19 vaccines continue to protect children from severe COVID-19 and MIS-C during the Omicron wave.

## Maternal COVID-19 vaccination

As of June 30, 2022, COVID-19 has been diagnosed in 220,673 pregnant people in the United States, resulting in 298 deaths (1). Pregnant people with COVID-19 are at increased risk of poor maternal and neonatal outcomes, and the CDC recommends vaccination for all pregnant people or those who plan to become pregnant. COVID-19 vaccines mitigate poor maternal outcomes; according to a study of 10,861 vaccinated pregnant people matched to an equal number of unvaccinated control pregnant individuals, the Pfizer-BioNTech vaccine was highly effective against COVID-19 and COVID-19–related hospitalization (17). In addition, maternal vaccination also confers protection for infants against COVID-19 and its complications. According to a multicenter study in the United States, the overall effectiveness of maternal vaccination against COVID-19–related hospitalization was 52% (95% CI, 33%–65%) (18). Interestingly, maternal vaccine effectiveness varied from 80% (95% CI, 60%–90%) during the Delta wave to 38% (95% CI, 8%–58%) during the Omicron wave (18).

## Concluding remarks

COVID-19 is a vaccine-preventable illness; everyone 6 months and older should get vaccinated. COVID-19 vaccines are safe and effective in children 6 months and older, and maternal vaccination confers protection for those under 6 months old. Following the most recent EUA amendment by the FDA, children of all ages now stand to benefit from either mRNA COVID-19 vaccine. The most crucial next step is to address parental vaccine hesitancy and encourage vaccine uptake. In addition, further research is necessary to determine the safety and effectiveness of COVID-19 vaccines in special populations, such as pediatric solid organ and stem cell transplant recipients, children with cancer, and children with other immunocompromising conditions. Moreover, additional studies are needed to determine the optimal booster strategy in children, especially in the context of emerging SARS-CoV-2 variants of concern.

## Figures and Tables

**Figure 1:**
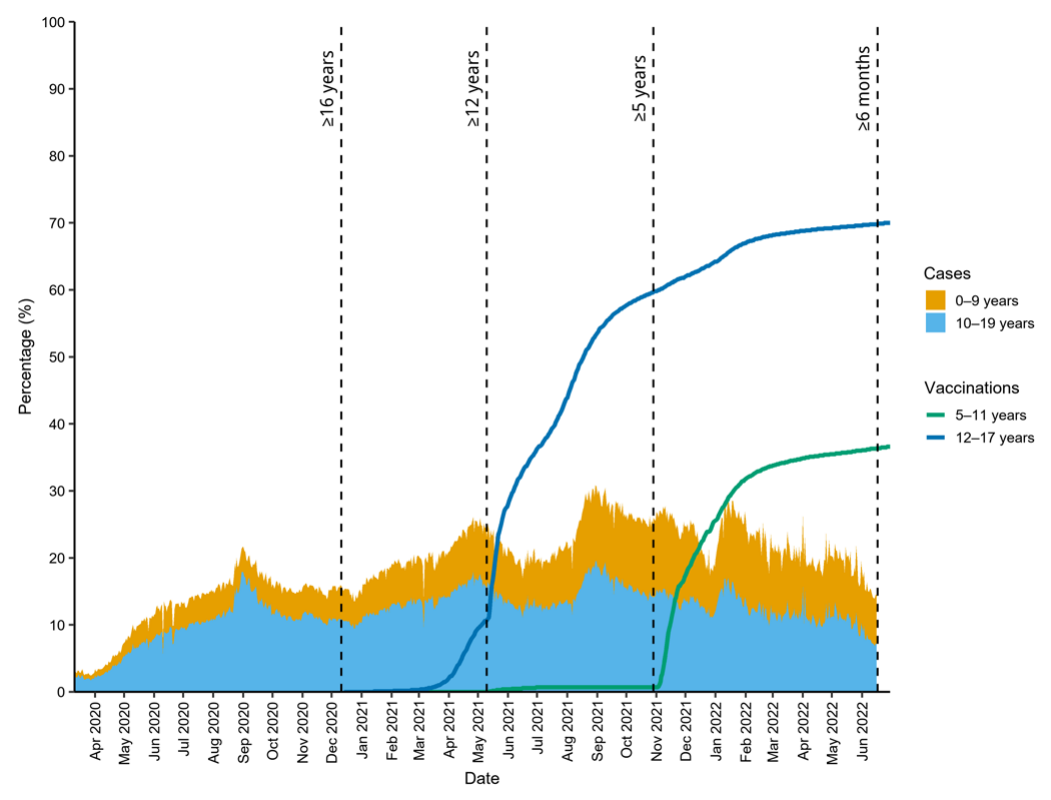
Stacked area plot of the proportion of COVID-19 cases diagnosed in individuals 0–19 years old (of all COVID-19 cases in the United States). Superimposed are line plots of the proportions of US children and adolescents 5–11 and 12–17 years old, respectively, who received at least one dose of vaccine. The dashed vertical lines indicate the dates the Emergency Use Authorization was issued and subsequently amended (adjacent text indicates coverage; refs. 2–5) for the different age groups. Both data sets are freely available on the CDC website (19, 20).

## Version 1 09/01/2022

Electronic publication

## References

[1] https://covid.cdc.gov/covid-data-tracker/#.

[2] https://www.fda.gov/news-events/press-announcements/fda-takes-key-action-fight-against-covid-19-issuing-emergency-use-authorization-first-covid-19.

[3] https://www.fda.gov/news-events/press-announcements/coronavirus-covid-19-update-fda-authorizes-pfizer-biontech-covid-19-vaccine-emergency-use.

[4] https://www.fda.gov/news-events/press-announcements/fda-authorizes-pfizer-biontech-covid-19-vaccine-emergency-use-children-5-through-11-years-age.

[5] https://www.fda.gov/news-events/press-announcements/coronavirus-covid-19-update-fda-authorizes-moderna-and-pfizer-biontech-covid-19-vaccines-children.

[6] Galanis P (2022). Willingness, refusal and influential factors of parents to vaccinate their children against the COVID-19: a systematic review and meta-analysis. Prev Med.

[7] Gaythorpe KAM (2021). Children’s role in the COVID-19 pandemic: a systematic review of early surveillance data on susceptibility, severity, and transmissibility. Sci Rep.

[8] Halasa NB Life-threatening complications of influenza versus COVID-19 in U.S. children. Clin Infect Dis.

[9] Henderson LA (2020). American College of Rheumatology Clinical Guidance for multisystem inflammatory syndrome in children associated with SARS-CoV-2 and hyperinflammation in pediatric COVID-19: version 1. Arthritis Rheumatol.

[10] Nygaard U (2022). Incidence and clinical phenotype of multisystem inflammatory syndrome in children after infection with the SARS-CoV-2 delta variant by vaccination status: a Danish nationwide prospective cohort study. Lancet Child Adolesc Health.

[11] Zambrano LD (2022). Effectiveness of BNT162b2 (Pfizer-BioNTech) mRNA vaccination against multisystem inflammatory syndrome in children among persons aged 12-18 years — United States, July–December 2021. MMWR Morb Mortal Wkly Rep.

[12] Yousaf AR (2022). Reported cases of multisystem inflammatory syndrome in children aged 12-20 years in the USA who received a COVID-19 vaccine, December, 2020, through August, 2021: a surveillance investigation. Lancet Child Adolesc Health.

[13] Ouldali N (2022). Hyper inflammatory syndrome following COVID-19 mRNA vaccine in children: a national post-authorization pharmacovigilance study. Lancet Reg Health Eur.

[14] Price AM (2022). BNT162b2 protection against the omicron variant in children and adolescents. N Engl J Med.

[15] Cohen-Stavi CJ BNT162b2 vaccine effectiveness against omicron in children 5 to 11 years of age. N Engl J Med.

[16] Holm M Risk and phenotype of multisystem inflammatory syndrome in vaccinated and unvaccinated Danish children before and during the omicron wave. JAMA Pediatr.

[17] Dagan N (2021). Effectiveness of the BNT162b2 mRNA COVID-19 vaccine in pregnancy. Nat Med.

[18] Halasa NB (2022). Maternal vaccination and risk of hospitalization for Covid-19 among infants. N Engl J Med.

[19] https://data.cdc.gov/Case-Surveillance/COVID-19-Case-Surveillance-Public-Use-Data/vbim-akqf.

[20] https://data.cdc.gov/Vaccinations/COVID-19-Vaccination-and-Case-Trends-by-Age-Group-/gxj9-t96f.

